# Efficacy of intradermal acupuncture for ocular surface diseases secondary to refractory peripheral facial palsy: a randomized controlled trial study protocol

**DOI:** 10.3389/fmed.2026.1873689

**Published:** 2026-07-20

**Authors:** Jiayi Yao, Lusha Cen, Qiushuang Li, Shan Liu, Chenjia Zhu, Lihua Xuan, Binyan Yu

**Affiliations:** 1 Department of Acupuncture and Moxibustion, The First Affiliated Hospital of Zhejiang Chinese Medical University (Zhejiang Provincial Hospital of Chinese Medicine), Hangzhou, China; 2 Department of Ophthalmology, The First Affiliated Hospital of Zhejiang Chinese Medical University (Zhejiang Provincial Hospital of Chinese Medicine), Hangzhou, China; 3 Department of Scientific Research, The First Affiliated Hospital of Zhejiang Chinese Medical University (Zhejiang Provincial Hospital of Chinese Medicine), Hangzhou, China

**Keywords:** intradermal acupuncture, ocular surface diseases, randomized, refractory peripheral facial palsy, study protocol

## Abstract

**Introduction:**

Refractory Peripheral Facial Palsy (RPFP) is defined as peripheral facial paralysis (PFP) that fails to achieve significant clinical recovery despite conventional therapeutic interventions. Due to severe facial nerve injury and a prolonged recovery course, RPFP often results in lagophthalmos, which in turn predisposes to ocular surface diseases (OSDs), most commonly exposure keratitis and dry eye disease. Currently, symptomatic and supportive therapies such as sodium hyaluronate eye drops are standard for managing OSDs secondary to RPFP, yet no other effective treatments have been established. This study aims to systematically evaluate the efficacy and safety of intradermal acupuncture (IA) as a potential therapeutic intervention for OSDs secondary to RPFP.

**Methods and analysis:**

This is a pilot, single-center, two-arm randomized controlled trial protocol to be conducted at The First Affiliated Hospital of Zhejiang Chinese Medical University. A total of 78 eligible participants will be randomly allocated to the intradermal acupuncture group (IA group, *n* = 39) or the sham acupuncture group (SIA group, *n* = 39) at a 1:1 ratio. Both groups will receive a 4-week intervention period followed by a 6-week follow-up phase. Participants in the IA group will receive IA treatment, while those in the SIA group will receive SIA treatment with placebo needles. During the treatment period, the WeChat mini-program “Facial Palsy Ocular Comfort Smart Assistant” will be employed to intelligently assist patients in performing self-administered IA pressing. The primary outcome measure is the proportion of participants with a ≥ 12-point reduction in Ocular Surface Disease Index (OSDI) score from baseline to 4-week intervention. Secondary outcome measures include ocular surface function assessments [Tear Meniscus Height (TMH), Non-invasive Tear Film Break-Up Time (NIBUT), Meibography], Paralytic Lagophthalmos Measurement (PLM), and Facial Nerve Grading Scale 2.0 (FNGS 2.0) score. This study aims to investigate the clinical efficacy of IA intervention for OSDs secondary to RPFP and explore an effective, convenient therapeutic strategy for OSDs secondary to RPFP.

**Clinical trial registration:**

ClinicalTrials.gov, NCT07537426.

## 1 Introduction

Peripheral facial palsy (PFP) is a common neurological disorder, with Bell’s palsy accounting for the largest proportion (1). Severe facial nerve injury or inadequate clinical intervention can lead to refractory peripheral facial palsy (RPFP) (2); its incidence can be as high as 40% (3). RPFP is characterized by unilateral orofacial deviation, paralytic lagophthalmos, ipsilateral facial synkinesis, and paradoxical facial movement. RPFP often has a poor prognosis and may result in persistent sequelae or permanent facial paralysis (3).

RPFP often causes ocular surface disorders (OSDs). Facial nerve injury can paralyze the orbicularis oculi muscle, preventing full eyelid closure for extended periods and leading to damage of the ocular surface (4). This damage worsens with the unavoidable use of electronic devices, which decreases blink rate and increases tear evaporation (5). A recent epidemiological study found that 68.3% of RPFP patients have superficial punctate keratopathy, 43.3% develop exposure keratopathy, and 85% show two or more concurrent OSD-related clinical features (6). This health issue greatly reduces patients’ ability to work and affects their quality of life.

No standardized definition of RPFP currently exists within the medical community (2). Electroneurography (ENoG) is widely used to predict PFP prognosis (7), and it records the compound muscle action potential (CMAP) amplitude ratio between the affected and unaffected sides (8). A CMAP amplitude ratio below 20% correlates with a minimum recovery period of 4 months, and most such patients show incomplete recovery (9). Therefore, this study enrolls PFP patients with a CMAP amplitude ratio < 20%. Notably, clinical guidelines lack explicit recommendations for diagnosing or managing OSDs secondary to RPFP.

Dry eye disease (DED) assessment tools are suitable for quantifying OSD severity in RPFP. Although the primary etiologies differ, OSDs secondary to RPFP and DED share extensive pathophysiological overlap, including tear film instability (10), ocular surface inflammation (4), and meibomian gland dysfunction (11). Accordingly, we adopted DED outcome measures, including the Ocular Surface Disease Index (OSDI), tear meniscus height (TMH), non-invasive tear film break-up time (NIBUT), and meibography.

Current treatment for OSDs secondary to RPFP relies on supportive care, such as sodium hyaluronate eye drops, with surgery considered only when necessary (12). However, prolonged corneal exposure and frequent use of electronic devices often fail to achieve optimal clinical outcomes (13). Therefore, there is a need for a safe, effective, and convenient alternative therapy. Acupuncture is a well-established treatment for PFP and is recommended by the World Health Organization (14). Nevertheless, the efficacy of conventional acupuncture in RPFP is limited by operator skill, visit frequency, and the short duration of effect from a single session.

As an innovative needling technique, IA delivers sustained and low-intensity stimulation, overcoming the limitations of conventional acupuncture. A prior study demonstrates that IA is comparably effective to conventional acupuncture in alleviating moderate-to-severe dry eye symptoms (15). Considering the similar pathological mechanisms between DED and OSDs secondary to RPFP, we hypothesize that IA may also be effective for OSDs secondary to RPFP.

Accordingly, we designed a randomized, sham-controlled trial to rigorously evaluate the efficacy and safety of IA for OSDs secondary to RPFP, with the aim of establishing a broadly applicable clinical strategy for this unmet need.

## 2 Study design and methods

### 2.1 Design and setting

**Figure 1:**
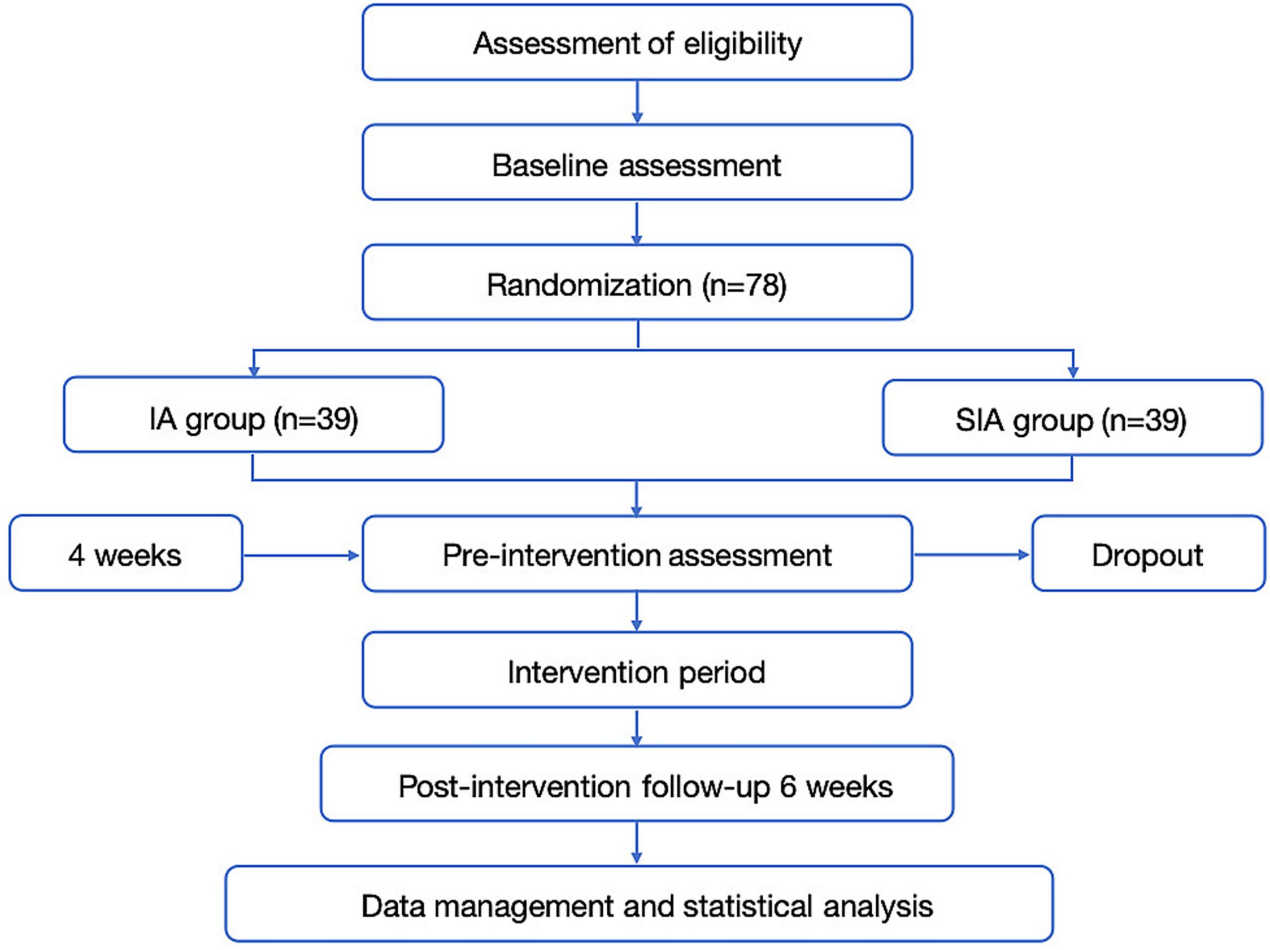
Flowchart of the research procedure.

This trial will be conducted at the First Affiliated Hospital of Zhejiang Chinese Medical University. The trial was approved and regularly reviewed by the hospital’s research ethics committees. The trial began in April 2026 and is expected to end in March 2028. The intervention includes a 4-week phase followed by a 6-week follow-up, involving either IA or SIA treatment. The trial flow diagram is illustrated in Figure 1, and the enrollment schedule is illustrated in Table 1.

**Table 1:** The chart of enrollment, interventions, and assessments.

Timepoint	Study period						
	Enrollment	Allocation	Treatment		Follow-up		
	Week 1	Week 0	Week 2	Week 4	Week 6	Week 8	Week 10
Enrolment							
Eligibility screen	×						
Informed consent	×						
Basic information	×						
Medical history	×						
Randomization		×					
Interventions							
The IA Group (*n* = 39)		×	×	×			
The SIA Group (*n* = 39)		×	×	×			
Assessments							
ENoG	×						
FNGS 2.0, OSDI, PLM	×		×	×	×	×	×
TMH, NIBUT, Meibography	×			×			×
Participants safety							
Adverse events	×	×	×	×	×	×	×
Blinding evaluation							
Bang Blinding Index				×			
Compliance monitoring							
The pressing completion rate				×			

### 2.2 Participants and informed consent

Participants are recruited from the outpatient clinics of the acupuncture and moxibustion department. Within each severity level, those who consent and meet the criteria are randomly assigned in a 1:1 ratio to receive either IA or SIA over a 4-week period, alongside a standardized background treatment. All prospective participants will be given detailed information about the study goals, procedures, potential benefits, and risks. Written informed consent is voluntarily obtained from each individual prior to enrollment, and participants reserve the right to withdraw from the study at any time.

Participants meeting the following criteria will be included: (1) Male or female patients aged 18–65 years; (2) Clinical diagnosis of PFP with disease duration of 1 month to 1 year; (3) FNGS 2.0 score ≥15 points; (4) ENoG shows a CMAP amplitude ratio ≤20%; (5) At least one self-reported ocular symptom (such as dryness, foreign body sensation, fatigue and other discomfort or vision problems) together with an OSDI score falling within the range of 30 to 80.

Participants will be excluded if they have any of the following conditions: (1) Other eye diseases such as glaucoma, keratitis, retinopathy, or acute inflammation of the conjunctiva, sclera, or cornea; (2) Recent intraocular surgery or laser therapy within the last 90 days; (3) Use of systemic or topical antibiotics or tear-affecting medications within the past 3 weeks, or dry eye treatments within 2 weeks; (4) Lacrimal passage obstruction, dacryocystitis, punctal occlusion, or neurological disorders other than PFP that prevent full eyelid closure; (5) Coagulation disorders, open wounds, or local infections at the treatment sites; (6) Allergy to IA materials such as stainless steel or adhesive tape; (7) Pregnancy or breastfeeding; (8) Severe cardiac, liver, kidney, psychiatric conditions, or malignant tumors; (9) Participation in another clinical trial within the last month. The basic information and clinical data of each participant will be recorded using a case report form. The independent researcher will enter the data into an electronic data capture (EDC) system in a timely manner.

### 2.3 Randomization and allocation concealment

Randomization is carried out using the hospital’s central system, employing a block randomization design with stratification based on age and disease duration to ensure balanced group allocation. Eligible participants are randomly assigned in a 1:1 ratio to receive either IA or SIA treatment. An independent statistician, who takes no part in participant recruitment, treatment delivery, or outcome evaluation, generates and protects the random allocation sequence. Group assignments are placed in sequentially numbered opaque envelopes, which remain sealed until after written informed consent has been obtained. This procedure ensures proper allocation concealment.

### 2.4 Blinding

This is a participant-blinded, outcome assessor-blinded, and statistician-blinded sham-controlled trial. Due to the nature of the acupuncture intervention, treating practitioners cannot be blinded and are required to perform either IA or SIA.

Specifically, participants are blinded to group assignment as the two types of needles are identical; outcome assessors and the statistician are blinded to allocation and not involved in treatment delivery or interim analyses; treating practitioners are unblinded but take no part in outcome assessment or data analysis.

To preserve blinding integrity, participants in the IA and SIA groups are treated in separate, partitioned clinical rooms to prevent intergroup communication. All investigators adhere to strict role separation protocols. Following the 4-week intervention, a formal blinding evaluation will be conducted to assess participants’ perceived treatment assignment, and the Bang Blinding Index will be calculated.

### 2.5 Intervention

All participants will receive a standardized background treatment regimen throughout the full 10-week study period. During the 4-week intervention period, they will receive either IA or SIA, according to their assigned treatment allocation.

#### 2.5.1 IA group

**Figure 2:**
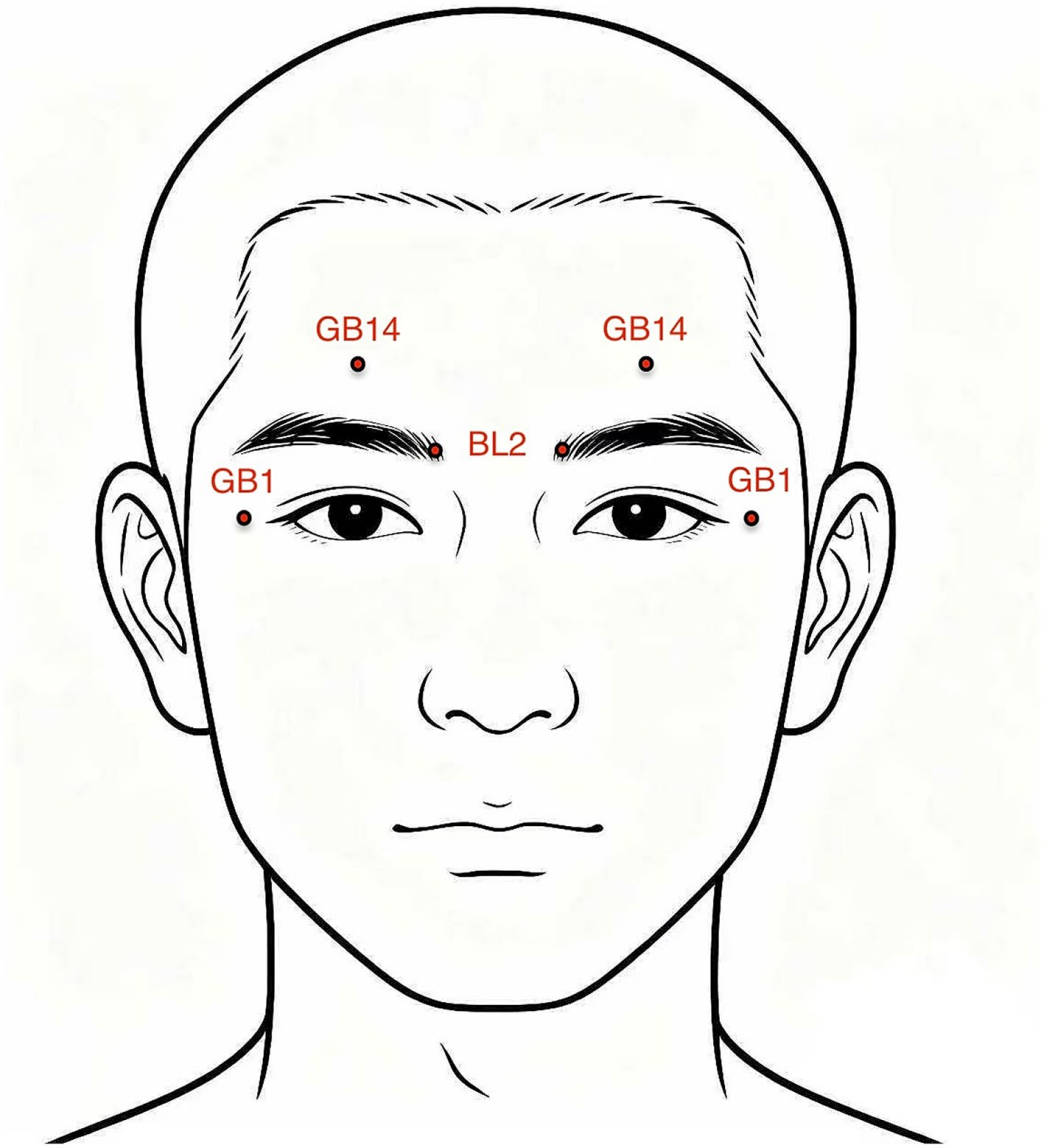
Locations of selected acupuncture points.

**Figure 3:**
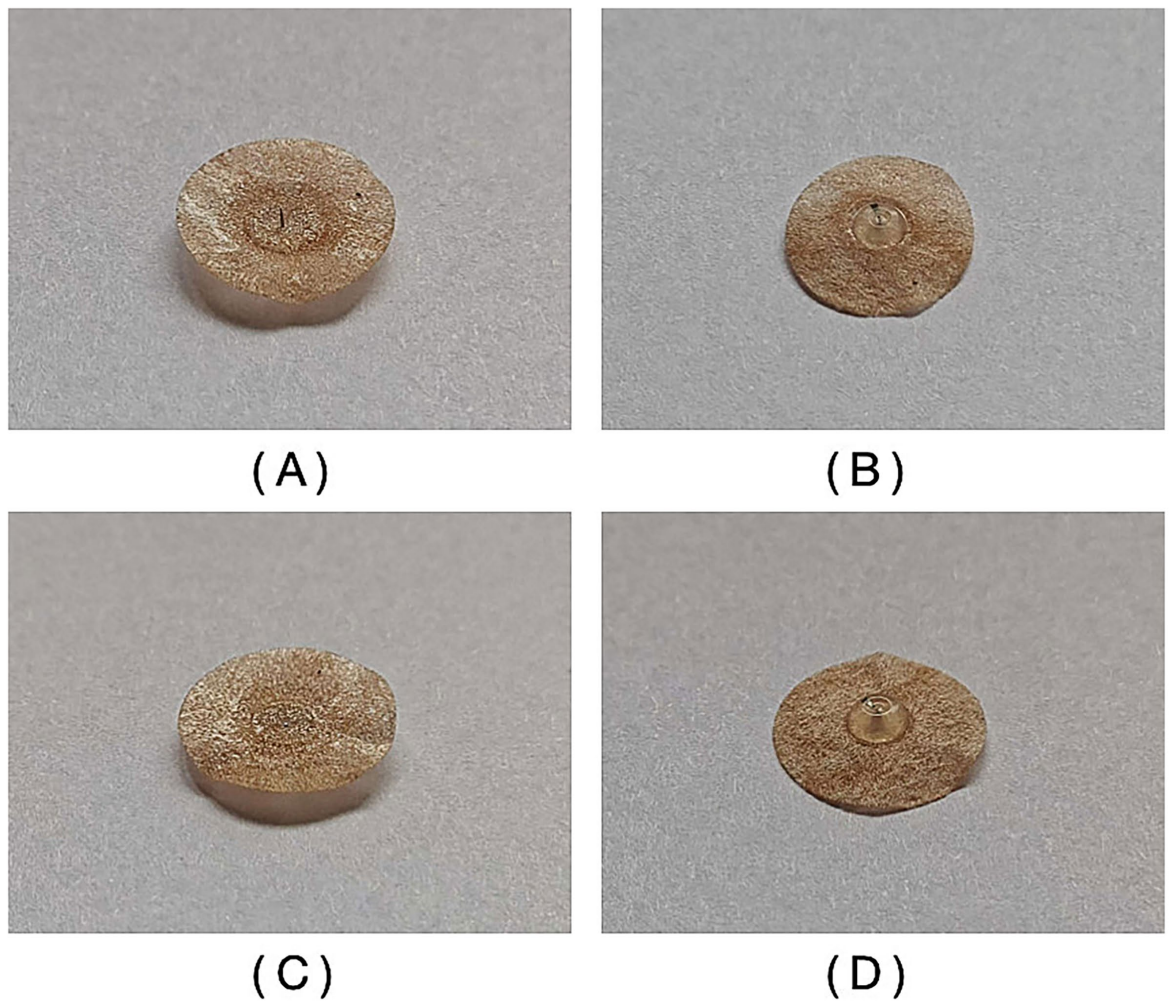
The appearance of IA and SIA needles. (**A**: inner surface of IA; **B**: outer surface of IA; **C**: inner surface of SIA; **D**: outer surface of SIA).

IA treatment is applied to the bilateral acupoints GB14, BL2, and GB1, as illustrated in Figure 2. The IA needles’ object pictures, as illustrated in Figures 3A,B. Under strict aseptic conditions, sterile IA needles (0.20 × 1.2 mm; Seirin PYONEX; Seirin Corporation, Shizuoka, Japan) are inserted perpendicularly into the skin and retained *in situ*. Each needle remains in place for 72 h, with replacements performed twice weekly. The total intervention period lasts 4 weeks. During the IA intervention, participants are instructed to perform standardized, self-administered acupoint pressing using a dedicated WeChat mini-program. The pressing regimen consists of three sessions per day, each lasting 3 min, at a frequency of 60 presses per minute. Participants are encouraged to apply as much pressure as tolerated.

#### 2.5.2 SIA group

The SIA needles (Seirin PYONEX; Seirin Corporation, Shizuoka, Japan) are identical to the IA needles in size and color. They feature a thin silicone pad in place of the needle body, as illustrated in Figures 3C,D. These needles are used on the same acupoints and left in place for 72 h. And the WeChat mini-program is also used for standardized self-pressing. The pressing regimen and acupoint selection are consistent with those in the IA group. A total of eight treatment sessions are conducted over 4 weeks.

#### 2.5.3 Standardized background treatment

All participants receive the same standard background treatment, consisting of conventional acupuncture for PFP and ocular surface supportive care. In this trial, IA and SIA are evaluated as add-on interventions to the standardized background treatment, rather than as stand-alone therapies.

Before each IA or SIA session, all participants receive conventional acupuncture. They are placed in a supine position. After applying routine skin disinfection, sterile and single-use acupuncture needles are inserted into predetermined acupoints: the affected side’s BL2, GB1, GB14, ST2, SI18, ST4, ST6, ST7, SJ17, EX-HN5, and EX-HN16, along with bilateral LI4, as detailed in Table 2.

**Table 2:** Locations of acupuncture points.

Acupoints	Location
BL2 (Cuanzhu)	In the depression at the medial end of the eyebrow.
GB1 (Tongziliao)	In the depression 0.5 cun lateral to the outer canthus of the eye.
GB14 (Yangbai)	1 cun superior to the eyebrow, directly above the center of the pupil.
ST2 (Sibai)	In the infraorbital foramen.
SI18 (Quanliao)	In the depression directly inferior to the outer canthus of the eye, at the inferior border of the zygomatic bone.
ST4 (Dicang)	0.4 cun lateral to the angle of the mouth.
ST6 (Jiache)	One fingerbreadth anterosuperior to the angle of the mandible.
ST7 (Xiaguan)	In the depression between the midpoint of the inferior border of the zygomatic arch and the mandibular notch.
SJ17 (Yifeng)	In the depression anterior to the inferior end of the mastoid process.
EX-HN5 (Taiyang)	One fingerbreadth posterior to the midpoint between the lateral end of the eyebrow and the outer canthus.
EX-HN16 (Qianzheng)	0.5–1 cun anterior to the auricular lobe.
LI4 (Hegu)	On the radial side of the midpoint of the second metacarpal bone.

Following needle insertion, electroacupuncture (EA) stimulation is applied using an intermittent waveform at 40 Hz. The stimulation intensity is adjusted according to each participant’s tolerance level. Treatment is administered twice weekly, with each session lasting 30 min. The entire treatment course spans 10 weeks. No adjunctive therapies are provided to participants during the treatment period.

Ocular surface supportive care is provided using 0.1% sodium hyaluronate eye drops, administered as one drop in each eye, three times daily, for 10 weeks.

During the study period, no adjunctive therapies other than the standardized background treatment described above are permitted. Any additional treatments received by participants—such as other eye drops, systemic medications affecting tear secretion, or other acupuncture modalities—will be documented as concomitant medications and adjusted for in the analyses.

#### 2.5.4 Compliance monitoring

A dedicated WeChat mini-program (“Facial Palsy Ocular Comfort Smart Assistant”) monitors patient adherence to self-administered acupoint pressing. The mini-program provides standardized operational guidance (instructional videos and written descriptions specifying pressing frequency, duration, intervals, and needle retention time), intelligent scheduled reminders (three sessions per day with a 4-h interval), a pressing log and a simplified symptom-recording module (automatically recording session duration, timestamps, and completion, while allowing patients to report ocular symptoms), and an adverse event reporting module (immediately notifying the study physician of any intervention-related discomfort). The prescribed total number of pressing sessions is 84 over the 4-week intervention. A session is considered valid if the actual pressing duration is ≥2 min 45 s, and the primary compliance metric is the completion rate (valid sessions/84 × 100%). Based on this rate, patients are categorized as good adherers (≥80%), moderate adherers (50–79%), or poor adherers (<50%).

### 2.6 Outcome measures

#### 2.6.1 Primary outcome

The primary outcome measures the proportion of participants who show a reduction of ≥12 points on the OSDI from baseline to the end of the 4-week intervention. This assessment is conducted at enrollment (week −1) and during treatment visits at weeks 2, 4, 6, 8, and 10.

OSDI captures subjective DED symptoms via a 12-item questionnaire with scores ranging from 0 to 100. For this study, the validated Chinese version of the OSDI (C-OSDI) is applied (16). The OSDI captures patient-reported symptoms (dryness, irritation, and visual fluctuations) (17), which are equally common in patients with facial palsy (18). According to previous studies (19), the minimal clinically important difference (MCID) for the OSDI ranges from 7.3 to 13.4 points in patients with severe dry eye, with a generally accepted MCID of 10 points (20).

Furthermore, based on the pre-test results of the present study, a 12-point reduction is considered indicative of “significant improvement in patient perception.” Therefore, a decrease in the OSDI score of ≥12 points from baseline after a 4-week intervention is considered indicative of effective treatment.

#### 2.6.2 Secondary outcome measures

##### 2.6.2.1 Ocular surface function assessments (TMH, NIBUT, and Meibography)

TMH, NIBUT, and Meibography are measured using a comprehensive ocular surface analyzer (Gaush iDea FOS) in different measurement modes, performed at enrollment (week −1) and at treatment visits at weeks 4 and 10.

TMH represents the basal tear volume along the lower eyelid margin, with a diagnostic cutoff of ≤0.2 mm for dry eye (21). Non-invasive measurement of TMH is performed at the central portion of the lower eyelid margin. For each eye, three consecutive readings are recorded, and the mean value is used for analysis. A decrease in TMH suggests insufficient tear secretion or increased evaporation (15). NIBUT measures the time from when the eyelids are fully open after blinking until the tear film first breaks. Three consecutive readings are taken and averaged. It directly evaluates tear film stability and the extent of ocular surface injury (22). Meibography evaluates the morphology and degree of meibomian gland loss. Utilizing the infrared mode on the comprehensive ocular surface analyzer (Gaush iDea FOS), images of both the upper and lower eyelids are obtained. The degree of gland loss and structural changes is graded using a standardized meiboscore system, usually from grade 0 (no loss) to grade 3 (severe loss, with >2/3 of the gland area affected) (23).

##### 2.6.2.2 PLM

PLM is quantified with a precise digital caliper by measuring the palpebral fissure width at the mid-pupillary line, with the patient in primary gaze. Examination results vary with the severity of lagophthalmos and its duration persists (24). Measurements of the affected eye will be taken under two standardized conditions: spontaneous, relaxed eyelid closure and maximal forced eyelid closure. Each reading will record the vertical gap of the palpebral fissure in millimeters, reflecting the extent of incomplete eyelid contact during natural orbicularis oculi relaxation and maximum voluntary contraction, respectively. These measurements will be recorded at enrolment (week −1) and during treatment visits at weeks 2, 4, 6, 8, and 10.

##### 2.6.2.3 FNGS 2.0

FNGS 2.0 is a standardized instrument for the quantitative assessment of facial nerve dysfunction (25), enabling objective evaluation through region-specific scores. The examiner subjectively rates the function of four discrete facial regions: the brow, eye, nasolabial fold, and oral commissure. Each regional score ranges from 1 to 6, and higher scores indicate worse function. The severity of synkinetic movements is rated on a scale from 0 to 3. The final composite score is calculated by summing the four regional scores and the synkinesis severity score; a higher total indicates more severe facial nerve impairment (9). This assessment is conducted at enrollment (week −1) and at treatment visits at weeks 2, 4, 6, 8, and 10.

### 2.7 Safety evaluation

Adverse events (AEs) encompass bleeding, haematoma, dizziness, local pain, acupuncture-related infections, and eye drop-induced adverse reactions (e.g., ocular irritation, conjunctival congestion, and allergic responses). At the time of onset, all AEs are recorded in detail on the Case Report Form (CRF). Serious adverse events (SAEs)—defined as death, life-threatening conditions, disability, or hospitalization—require reporting by the principal investigator to the ethics committees of both the trial site and the hospital within 24 h, alongside accurate CRF documentation. All AEs are followed until their resolution. If necessary, the ethics committee decides whether the trial should be discontinued.

### 2.8 Sample size

This study is designed as a superiority trial. Sample size calculations are performed using PASS V2025 based on superiority testing. According to our pilot data, the expected response rate is 30% in the SIA group (P_2_ = 0.30) and 75% in the IA group (P_1_ = 0.75). A clinically meaningful superiority margin of 0.1 is prespecified. To control for type I error due to multiple comparisons, a two-sided significance level of 0.0125 (adjusted from the original *α* = 0.05) is used. Power is set at 80%. Under these assumptions, the required sample size is 31 participants per group, achieving a power of approximately 80% (≥0.8). Accounting for an anticipated dropout rate of approximately 20% and a 1:1 allocation ratio, at least 39 participants are needed per group, giving a total minimum of 78 participants.

### 2.9 Statistical analysis

All analyses adhere to a pre-specified statistical analysis plan (SAP) and are conducted using SAS 9.4. The intention-to-treat (ITT) population serves as the primary analysis set, while the per-protocol (PP) population is used for supportive analyses; a safety set comprises all treated participants. Baseline characteristics are summarized using standardized mean differences (SMDs). For the primary outcome, a mixed-effects logistic regression model is employed, with treatment arm (IA and SIA) as the fixed effect, adjusted for baseline OSDI score and stratification factors (age and disease duration). The treatment effect will be expressed as an odds ratio (OR) with its 95% Confidence Interval (CI). Continuous secondary outcomes are assessed using analogous models; ordered categorical endpoints use cumulative-link mixed models; dichotomous endpoints employ mixed-effects logistic regression. Multiple comparisons are controlled via a prespecified hierarchical procedure for key secondary endpoints and the Benjamini–Hochberg method (q = 0.05) for others. Incomplete observations are processed via maximum likelihood in mixed models, with imputation (*m* = 50) used as a robustness assessment. AEs are summarized per group, providing risk estimates and 95% CIs. A blinded data review is performed prior to database lock. Effect estimates are presented alongside 95% CIs. Two-sided tests are used throughout, with statistical significance defined as *p* < 0.05. A detailed SAP is finalized before the database lock.

## 3 Discussion

RPFP often leads to persistent ocular surface damage due to prolonged lagophthalmos, reduced blink frequency, and impaired tear distribution (26). Common symptoms include eye discomfort, a foreign body sensation, photophobia, blurred vision, and even vision loss (24). Severe cases may develop exposure keratopathy, corneal ulceration, or even permanent visual impairment (10, 27). This refractory nature and the risk of permanent sequelae highlight the need for more effective treatments.

There are no direct studies on acupuncture or IA specifically for OSDs secondary to RPFP; however, indirect evidence provides some support. In DED, a previous study found that IA is similarly effective as traditional acupuncture in relieving moderate to severe dry eye symptoms (15), probably by influencing tear production, reducing inflammation, and regulating nerve function. In PFP, acupuncture is commonly used to treat PFP and has shown significant effectiveness and safety in clinical practice (14). The underlying mechanisms of acupuncture for PFP include reducing inflammation, increasing neurotrophic factors, and enhancing microcirculation in the face (28). These mechanisms may help confirm that IA has the potential to improve OSDs secondary to RPFP.

Thus, despite the absence of direct literature, the combination of evidence from DED and facial palsy supports our hypothesis. Meanwhile, both DED and OSDs secondary to RPFP converge on final common pathways. Based on the above evidence, we designed this experiment to assess the effectiveness of IA and validate our hypothesis.

Our research group’s preliminary pilot studies indicate that IA can effectively reduce symptoms of OSDs secondary to RPFP. The acupoints used in this study are GB14, BL2, and GB1. These acupoints are associated with the periorbital region and the facial nerve’s distribution. Continuous stimulation is believed to have a synergistic effect on facial nerve recovery and to improve ocular surface function. Additionally, a dedicated WeChat mini-program called the “Facial Palsy Ocular Comfort Smart Assistant” has been developed to standardize patient-administered acupoint pressing, enabling them to perform regular self-treatment at home.

This study adheres to strict clinical trial principles to ensure the scientific validity and reliability of the results. To minimize the placebo effect and subjective bias, a sham-controlled setup was used, involving IA needles and SIA needles that look identical. Consistent with protocols from prior authoritative research, this study employed the same type of SIA needles, whose design, preparation, and validity have been confirmed through extensive clinical use (29, 30).

Several limitations are present in this study. First, as a single-center trial, all participants were recruited exclusively from The First Affiliated Hospital of Zhejiang Chinese Medical University, which may introduce selection bias and limit the generalizability of the results to the broader population. Confirmation of these findings requires multi-center trials that include more heterogeneous patient Second, because of the nature of the acupuncture intervention, a double-blind design is impossible. The single-blind approach might introduce operator bias, and there’s a risk of unblinding for both IA and SIA treatments during the study. Third, evidence shows that simple manual self-pressing can stimulate acupoints and alleviate symptoms (31), which may confound the assessment of the actual therapeutic effects. Fourth, the 4-week intervention and 6-week follow-up are relatively brief. Therefore, future investigations should adopt longer observation periods to more precisely assess the sustained efficacy and durability of IA treatment for this condition.

In conclusion, this study aims to provide high-quality clinical evidence on the efficacy and safety of IA for OSDs secondary to RPFP, while also presenting a new, safe, convenient, and easily promotable treatment option for this condition. This intervention employs a doctor–patient cooperation approach that not only standardizes treatment but also facilitates home-based rehabilitation. This effectively alleviates the medical burden on both patients and healthcare providers. Consequently, IA shows promising potential for clinical use and wider adoption in managing OSDs secondary to RPFP.
